# Potential for the anaerobic oxidation of benzene and naphthalene in thermophilic microorganisms from the Guaymas Basin

**DOI:** 10.3389/fmicb.2023.1279865

**Published:** 2023-09-29

**Authors:** Hanna Zehnle, Carolin Otersen, David Benito Merino, Gunter Wegener

**Affiliations:** ^1^Max Planck Institute for Marine Microbiology, Bremen, Germany; ^2^MARUM, Center for Marine Environmental Sciences, University of Bremen, Bremen, Germany; ^3^Faculty of Geosciences, University of Bremen, Bremen, Germany

**Keywords:** aromatic hydrocarbon, thermophile, Guaymas Basin, *Desulfatiglans*, hydrocarbon degradation

## Abstract

Unsubstituted aromatic hydrocarbons (UAHs) are recalcitrant molecules abundant in crude oil, which is accumulated in subsurface reservoirs and occasionally enters the marine environment through natural seepage or human-caused spillage. The challenging anaerobic degradation of UAHs by microorganisms, in particular under thermophilic conditions, is poorly understood. Here, we established benzene- and naphthalene-degrading cultures under sulfate-reducing conditions at 50°C and 70°C from Guaymas Basin sediments. We investigated the microorganisms in the enrichment cultures and their potential for UAH oxidation through short-read metagenome sequencing and analysis. Dependent on the combination of UAH and temperature, different microorganisms became enriched. A Thermoplasmatota archaeon was abundant in the benzene-degrading culture at 50°C, but catabolic pathways remained elusive, because the archaeon lacked most known genes for benzene degradation. Two novel species of Desulfatiglandales bacteria were strongly enriched in the benzene-degrading culture at 70°C and in the naphthalene-degrading culture at 50°C. Both bacteria encode almost complete pathways for UAH degradation and for downstream degradation. They likely activate benzene via methylation, and naphthalene via direct carboxylation, respectively. The two species constitute the first thermophilic UAH degraders of the Desulfatiglandales. In the naphthalene-degrading culture incubated at 70°C, a Dehalococcoidia bacterium became enriched, which encoded a partial pathway for UAH degradation. Comparison of enriched bacteria with related genomes from environmental samples indicated that pathways for benzene degradation are widely distributed, while thermophily and capacity for naphthalene activation are rare. Our study highlights the capacities of uncultured thermophilic microbes for UAH degradation in petroleum reservoirs and in contaminated environments.

## Introduction

Aromatic hydrocarbons (AHs) are a naturally abundant group of hydrocarbons. They are highly hydrophobic and extremely stable molecules because of their planar conformation consisting of one or more six-carbon ring systems stabilized by delocalized π electrons (Aihara, 1992). AHs constitute a major part (20–60%) of petroleum and fossil fuel, and are thus naturally abundant in subsurface petroleum reservoirs (Gibson, 1975; Pevneva et al., 2017). On Earth’s surface, AHs originate mainly from incomplete combustion of fossil fuel (Blumer, 1976; Lima et al., 2005), from natural seepage on the ocean floor, and from accidental spillage during oil reservoir exploration or transport (Kawka and Simoneit, 1990; Wang et al., 1999).

The study of AH biodegradation is of high interest, because AHs are highly toxic and thus their release into the environment is associated with health hazards (WHO Regional Office for Europe, 2000). Moreover, it is important to understand dynamics of hydrocarbon degradation in deeply buried petroleum reservoirs. While AHs are toxic to various more complex life forms (Patel et al., 2020; Xu et al., 2020), microorganisms have developed metabolic pathways to degrade these compounds. Microbial degradation is the main mechanism of AH removal from the environment. AH degradation pathways differ fundamentally in the presence and absence of molecular oxygen (Alexander, 1981; Parales et al., 2002; Parales and Haddock, 2004; Fuchs et al., 2011). Under oxic conditions, many bacteria and some halophilic archaea degrade AHs rapidly after activation via oxygenases (Bugg, 2003; Fahy et al., 2006; Tapilatu et al., 2010; Erdogˇmuş et al., 2013). After ring cleavage, products converge as central intermediates like acetate, pyruvate, and succinate, which are shuttled into biomass production or enter central metabolic pathways (Fuchs, 1999). Anaerobic AH degradation yields less energy and sustains slower growth rates, therefore cultivation of the respective organisms is challenging. Still, successful enrichment or pure cultures have been obtained, which have allowed insights into the mechanisms of anaerobic AH degradation. Unsubstituted AHs (UAHs), i.e., AHs without functional groups, are especially challenging to degrade and the rate-limiting step is the initial activation of the very stable aromatic ring system (Heider, 2007).

Under anoxic conditions, benzene, the smallest UAH, is oxidized by mesophilic bacteria under sulfate-, nitrate-, iron-, and manganese-reducing and in syntrophic consortia with methanogenic archaea (Musat and Widdel, 2008; Villatoro-Monzón et al., 2008; Zhang et al., 2012; Atashgahi et al., 2018; Toth et al., 2021). While methylation to toluene and hydroxylation to phenol have been proposed as activation mechanisms, carboxylation to benzoate has become the favored pathway (Ulrich et al., 2005; Abu Laban et al., 2009; Zhang et al., 2013; Eziuzor et al., 2022). Enzymes for direct methylation or hydroxylation of benzene are currently unknown, but an anaerobic benzene carboxylase (AbcAD) belonging to the UbiD/UbiX-type carboxylases was identified in iron-reducing enrichment cultures of *Peptococcaceae* bacteria (Abu Laban et al., 2010; Luo et al., 2014). All activation pathways converge in the central intermediate benzoyl-CoA (BCoA; Porter and Young, 2014). Then, the aromatic ring system becomes dearomatized by ATP-dependent Class I benzoyl-CoA reductase (BCR) or ATP-independent class II BCR (Boll et al., 1997, 2000; Song and Ward, 2005; Wischgoll et al., 2005; Porter and Young, 2014; Huwiler et al., 2019). Subsequent ring fissure occurs via *Thauera* type or *Rhodopseudomonas* type ring hydrolysis (Harwood et al., 1998; Carmona et al., 2009; Porter and Young, 2014). A modified *β*-oxidation pathway, the lower BCoA pathway, yields acetyl-CoA (Carmona et al., 2009), which is shuttled into biomass production or completely oxidized to CO_2_ via the Wood-Ljungdahl (WL) or tricarboxylic acid (TCA) pathways (Krebs, 1954; Ragsdale, 1997).

Naphthalene, the next largest UAH, consists of two fused benzene rings. Several anaerobic bacteria oxidize naphthalene under sulfate-reducing (Galushko et al., 1999), iron-reducing (Kleemann and Meckenstock, 2011), and methanogenic (Christensen et al., 2004) conditions. The best-studied cultures are the pure culture of *Desulfatiglandaceae* bacterium NaphS2 and a highly enriched culture dominated (abundance >95%) by *Desulfobacterium* strain N47 (Galushko et al., 1999; Meckenstock et al., 2000; DiDonato et al., 2010; Selesi et al., 2010; Bergmann F. et al., 2011). Like for benzene, direct carboxylation is the likely activation mechanism for naphthalene in most cultures (Galushko et al., 1999; Musat et al., 2009; DiDonato et al., 2010; Kleemann and Meckenstock, 2011; Mouttaki et al., 2012; Kümmel et al., 2015). A gene cluster encoding a putative naphthalene carboxylase complex, including UbiD-like carboxylases similar to AbcA, has been described (Kümmel et al., 2015; Koelschbach et al., 2019; Heker et al., 2023). Subsequent degradation occurs via conversion to 2-naphthoyl-CoA (Bergmann F. D. et al., 2011; Meckenstock et al., 2016; Heker et al., 2023) and a three-step reductive dearomatization (Eberlein et al., 2013a,b; Estelmann et al., 2015; Meckenstock et al., 2016). A stepwise oxidation, which includes ring cleavage and removal of branched alkyl chains, produces acetyl-CoA, presumably by enzymes encoded in the *thn* operon which is found in NaphS2 and N47 (Meckenstock et al., 2016).

Temperature is an important factor for the rate of petroleum hydrocarbon degradation (Das and Chandran, 2011). While hydrocarbon biodegradation in petroleum reservoirs is assumed to take place up to 80–90°C (Wilhelms et al., 2001), most cultured anaerobic UAH degraders grow at around 30°C. The knowledge on thermophilic to hyperthermophilic UAH degraders is scarce. The combination of UAH degradation with sulfate reduction is of particular interest, because sulfate is an important electron acceptor introduced artificially into reservoirs during secondary oil recovery, thereby stimulating hydrocarbon degradation and reservoir souring (Marietou, 2021).

Here, we aimed to enrich UAH oxidizers operating under the least studied conditions: anaerobic metabolism, UAH degradation, and high temperatures. We used sediment from the hydrothermal vent site Guaymas Basin (GB) located in the Gulf of California (Mexico) for this endeavor. In the GB, petroleum-range hydrocarbons, including AHs, are naturally abundant (Bazylinski et al., 1988; Kawka and Simoneit, 1990). These hydrocarbons fuel microbial communities in anoxic upper sediment layers, which are characterized by steep thermal gradients that can reach ≥100°C at 30 cm depth (McKay et al., 2012, 2016; Teske et al., 2014). The conditions prevalent in the GB are rare in the accessible seafloor and resemble those of heated subsurface petroleum reservoirs (Pannekens et al., 2019). Therewith, the GB can be considered a surface analogue for petroleum reservoirs, which is well suited to study microbial processes like anaerobic AH degradation at high temperatures. In this study, we incubated hydrothermally heated GB sediment with different UAHs as electron donor and sulfate as electron acceptor at 50°C and 70°C.

## Materials and methods

### Anoxic cultivation

The push cores 4991-13 and 4991-14 used for anoxic cultivations were collected at the “Cathedral Hill” hydrothermal vent site with submersible *Alvin* during *RV Atlantis* cruise AT42-05 to the GB during dive 4,991 (27° 00′ 41.1″ N, 111° 24′ 16.3″ W, 2,013 m water depth, November 17, 2018). On the ship, the push cores were transferred to glass bottles, which were sealed with rubber stoppers, purged with argon and stored at 4°C. In the home laboratory, the cores were combined and mixed with anoxic sulfate-reducer medium (SRM; Laso-Pérez et al., 2018) in a ratio of 1:10 (v:v). The sediment slurry was distributed into autoclaved serum bottles in 100 mL aliquots. The bottles were sealed with butyl rubber stoppers. Benzene, naphthalene, phenanthrene, and pyrene were provided as sole electron and carbon donors. The UAHs were dissolved in silicone oil, which is non-biodegradable and was previously shown to decrease AH toxicity and aid in transport of AHs to microbial cells (Ye et al., 2019). 5 mL of the silicone oil-UAH mixture were added to the slurries, supplying a final UAH concentration of 10 mM. A negative control contained 5 mL silicone oil without substrate. The headspaces were filled with 2 atm N_2_:CO_2_ (90:10). Two temperature treatments, 50°C and 70°C, were applied, and bottles were incubated at gentle shaking (40 rpm) in the dark. For each substrate and each temperature, three replicates were prepared.

Sulfide production was assessed in bi-weekly intervals via a copper sulfate assay (Cord-Ruwisch, 1985) using increased sulfate reduction compared to the negative control as indicator for AH oxidation. Cultures were diluted 1:4 (v:v) in fresh SRM and supplied with fresh substrate when sulfide concentrations exceeded ~10 mM. To determine the doubling times of active microorganisms in the cultures, the sulfide production rates of cultures after the first dilution were used as a proxy, excluding dilutions with only two sulfide measuring points. Produced sulfide was displayed on a logarithmic (base 2) y-axis, and doubling times were calculated from the inclination m of exponential regression lines $y=n*e^{mx}$ with the equation:

$$\mathrm{Doubling}\;\mathrm{time}\;\left(d\right)=\frac{\ln\left(2\right)}{m}$$

### DNA extraction

After around 600 days of cultivation, cultures were sampled and DNA was extracted for metagenome sequencing. By this time, the active cultures had been diluted eight (benzene 50°C-B50), seven (benzene 70°C-B70), ten (naphthalene 50°C-N50), and two (naphthalene 70°C-N70) times. Of each culture, 40 mL were sampled, centrifuged (10 min, 3,100 ×*g*, 4°C) and the culture medium was discarded. DNA was extracted from the pellets using a modified SDS-protocol (Natarajan et al., 2016). DNA was also extracted in the same way from a 1 g pellet of dried sediment slurry (dry weight 202 mg mL^−1^) that was produced from the combined cores 4991-13 and 4991-14. The final DNA concentrations were determined in a fluorometric assay. DNA yields were 0.2 μg (B50), 3.4 μg (N50), 4.0 μg (B70), 0.6 μg (N70), and 0.7 μg (original sediment). Libraries were sequenced as 2 × 150 bp paired-end reads on an Illumina HiSeq3000 platform at the Max-Planck-Genome-Centre (Cologne, Germany). Between 4,142,459 (B70) and 4,247,237 (N70) raw reads were obtained.

### Short-read DNA analysis

Raw reads were quality-trimmed with BBDuk (included in BBMap version 38.79;^1^ minimum quality value: 20, minimum read length: 50; Bushnell, 2014). For the sediment slurry sample, the microbial community was estimated based on reconstructed small subunit (SSU) ribosomal RNA (rRNA) gene sequences mapped against the SILVA SSU reference database (version 138.1; Quast et al., 2013) with phyloFlash^2^ (Gruber-Vodicka et al., 2022). The trimmed reads of the culture samples were co-assembled with SPAdes (version 3.15.0;^3^ Bankevich et al., 2012). The output scaffolds were reformatted with anvi’o (version 7.1;^4^ Eren et al., 2015), simplifying names and excluding contigs <2,500 bp. The trimmed reads were then mapped to the reformatted scaffolds fasta using Bowtie 2 with local read alignment setting (version 2.4.2;^5^ Langmead and Salzberg, 2012). The output sequence alignment map (SAM) files were converted to binary alignment map (BAM) files with SAMtools (version 1.11;^6^ Danecek et al., 2021), which were indexed with anvi’o. A contigs database was created from the reformatted scaffolds file and profile databases were created for all samples with anvi’o. Open-reading frames (ORFs) of the contigs database, which anvi’o automatically identified using Prodigal (version 2.6.3;^7^ Hyatt et al., 2010), were annotated with the anvi’o-integrated databases NCBI clusters of orthologous genes (COGs; Tatusov et al., 1997), Kyoto Encyclopedia of Genes and Genomes (KEGG; Kanehisa et al., 2017), Protein Families (Pfams; Mistry et al., 2021), and KEGG orthologues HMMs (KOfams; Aramaki et al., 2020). Hidden Markov Model (HMM) searches for archaeal and bacterial single-copy core genes (SCGs) and genes encoding the dissimilatory sulfate reduction (DSR) pathway, which includes the three proteins sulfate adenylyltransferase (Sat), adenylylsulfate reductase (AprAB), and dissimilatory sulfite reductase (DsrAB), were run. Taxonomies were predicted for ORFs predicted for the contigs database with the Centrifuge classifier (version 1.0.2-beta;^8^ Kim et al., 2016). The profile databases were merged, enforcing hierarchical clustering. Metagenome-assembled genomes (MAGs) were created in the anvi’o interactive interface through manual binning. For this purpose, branches of the hierarchically clustered dendrogram were followed systematically in a counterclockwise direction, generating bins via clicking and observing the real-time statistics on completion and redundancy based on single-copy core genes (SCGs) were observed. All MAGs were then refined manually with anvi’o, using GC content, mean coverage in all samples, and gene taxonomy as guides. The quality of the final MAGs was determined with CheckM (version 1.1.3;^9^ Parks et al., 2015). Only MAGs with completion >50% and redundancy <10% after refinement were included in downstream analyses. Next, taxonomies were assigned to the MAGs using the GTDB toolkit GTDB-Tk (version 2.1.1;^10^ Chaumeil et al., 2020) and relative abundances of the MAGs in the samples were calculated with CoverM (version 0.6.1),^11^ which was run in genome mode. Prevalence of the MAGs in the original sediment was also estimated with CoverM using the trimmed read of the sediment slurry as input to map to the MAGs. The optimal growth temperature (OGT) was predicted for MAGs of interest with the OGT_prediction tool (version 1.0.3;^12^ Sauer and Wang, 2019) using the included regression models for Archaea and Bacteria which exclude genome size and 16S rRNA gene data. Average nucleotide identities (ANIs) between MAGs were determined with fastANI (version 1.33;^13^ Jain et al., 2018).

### Genome annotation

The COG, KEGG, Pfam, and KOfam annotations were exported for MAGs of interest with anvi’o. In addition, protein sequences of ORFs were extracted from these MAG with anvi’o. Amino acid sequences of genes involved in AH degradation, sulfate reduction and related genes (electron transfer, carbon fixation, cell appendage formation) from the domains *Archaea* and *Bacteria* were acquired from the National Center for Biotechnology Information (NCBI) Protein and the UniProtKB databases. For putative anaerobic benzene carboxylase (AbcAD), protein sequences were collected from recent publications (Abu Laban et al., 2010; Holmes et al., 2011; Luo et al., 2014). The nucleotide sequences presumably coding for AbcA and AbcD from Abu Laban et al. (2010) (GenBank accessions GU357992 and GU357991, respectively) were translated to amino acid sequences using the ExPASy translate tool.^14^ Additional amino acid sequences for subunits of class I BCRs (*bcrACD*/*bzdNQ*) amplified via PCR in the study by Song and Ward (2005), which have been deposited under GenBank accession numbers AY956841 to AY956907, were also acquired. Sequences for 3-hydroxypimeloyl-CoA dehydrogenase (*pimE*) and acetyl-CoA acyltransferase (*pimB*) were acquired from Atashgahi et al. (2018) (locus tags contig-100_24_2 and Contig-100_24_7, respectively). For each protein file, short sequences were removed with Seqtk (version 1.3).^15^ Local databases were created for the protein files with BLAST (version 2.10.1;^16^ Altschul et al., 1990). Amino acid sequences of the MAGs were compared to the local databases with BLASTp. BLASTp output was filtered with BLAST-QC (version 0.1;^17^ Torkian et al., 2020). Cutoff values for the identification of a given protein where: *e*-value <1e-10, identity ≥40%, and aligned length ≥ 80%. Proteins containing the heme-binding amino acid motif CxxCH (Bertini et al., 2006) were identified with a bash script in selected MAGs.

### Phylogenomic and genomic analysis of bacterial groups associated with enriched organisms

Two phylogenomic trees were constructed from concatenated alignments of single-copy core genes (SCGs). For the first tree, all 135 publicly available MAGs classified as order Desulfatiglandales in the Genome Taxonomy Database (GTDB) taxonomy tree^18^ (Parks et al., 2022) were downloaded from NCBI including metadata (Supplementary Table S1). Due to the high number of genomes, the Desulfatiglandales MAGs were dereplicated at species level (ANI ≥95%) prior to tree reconstruction with anvi’o, which uses fastANI, picking the MAG with highest similarity to all other MAGs of a species cluster as a representative. The phylogenomic tree was constructed with the 76 representative MAGs (Supplementary Table S2), the six Desulfatiglandales MAGs (5, 9, 34, 36, 46, 47) from this study, and five MAGs of the Desulfobulbia, a sister group of Desulfatiglandales, as outgroup. The second tree included all 26 publicly available MAGs assigned as order SZUA-161 of the class Dehalococcoidia in the GTDB taxonomy tree (Supplementary Table S3) downloaded from NCBI, including metadata, plus the SZUA-161 MAG of this study (MAG 33) and 10 MAGs of a sister order of SZUA-161 within Dehalococcoidia, UBA6952, as outgroup. For tree reconstruction, the selected MAGs were reformatted and a contigs database was created for each MAG with anvi’o (version 7.1). The anvi’o-integrated HMM collection was run on all contigs databases to identify bacterial SCGs, which were then aligned in a concatenated manner via anvi’o, which uses the multiple sequence alignment tool MUSCLE (version 5.1;^19^ Edgar, 2004). Trees were calculated with 30 SCGs using IQ-TREE (version 1.6.12;^20^ Nguyen et al., 2015). IQ-TREE was run using standard model selection followed by tree inference with 100 bootstrap iterations. Two MAGs (GCA_020351915.1 and GCA_020349865.1) and one MAG (GCA_020351795.1) were automatically excluded from the Desulfatiglandales and the SZUA-161 trees, respectively by IQ-TREE because they did not contain sufficient SCGs to infer meaningful phylogenies. Final trees were visualized with the Interactive Tree of Life (iTOL) online tool (version 6;^21^ Letunic and Bork, 2011). MAGs were compared via calculation of ANI with fastANI and amino acid identity (AAI) with the aai_wf workflow, which is part of the CompareM package (version 0.1.2).^22^

The optimal growth temperature (OGT) was predicted for the publicly available MAGs with the OGT_prediction tool (version 1.0.3). Key genes for anaerobic UAH/AH oxidation and downstream degradation were identified in Desulfatiglandales and SZUA-161 MAGs by running the previously built protein databases on the amino acid sequences of the MAGs in the same way and with the same selection criteria as described in the section “Genome annotation.”

### Data availability

The raw reads of short-read metagenome sequencing of the original sediment slurry and the four enrichment cultures have been deposited in the NCBI Sequence Read Archive (SRA) under BioProject PRJNA1013425 (accessions SRR25925499-SRR25925503).

### Code availability

The bash script used for the identification of CxxCH motifs in protein sequences of the MAGs is available under https://github.com/zehanna/UAH_oxidation.

## Results

### Thermophilic microorganisms from the GB degrade one- to two-ringed UAHs

**Figure 1:**
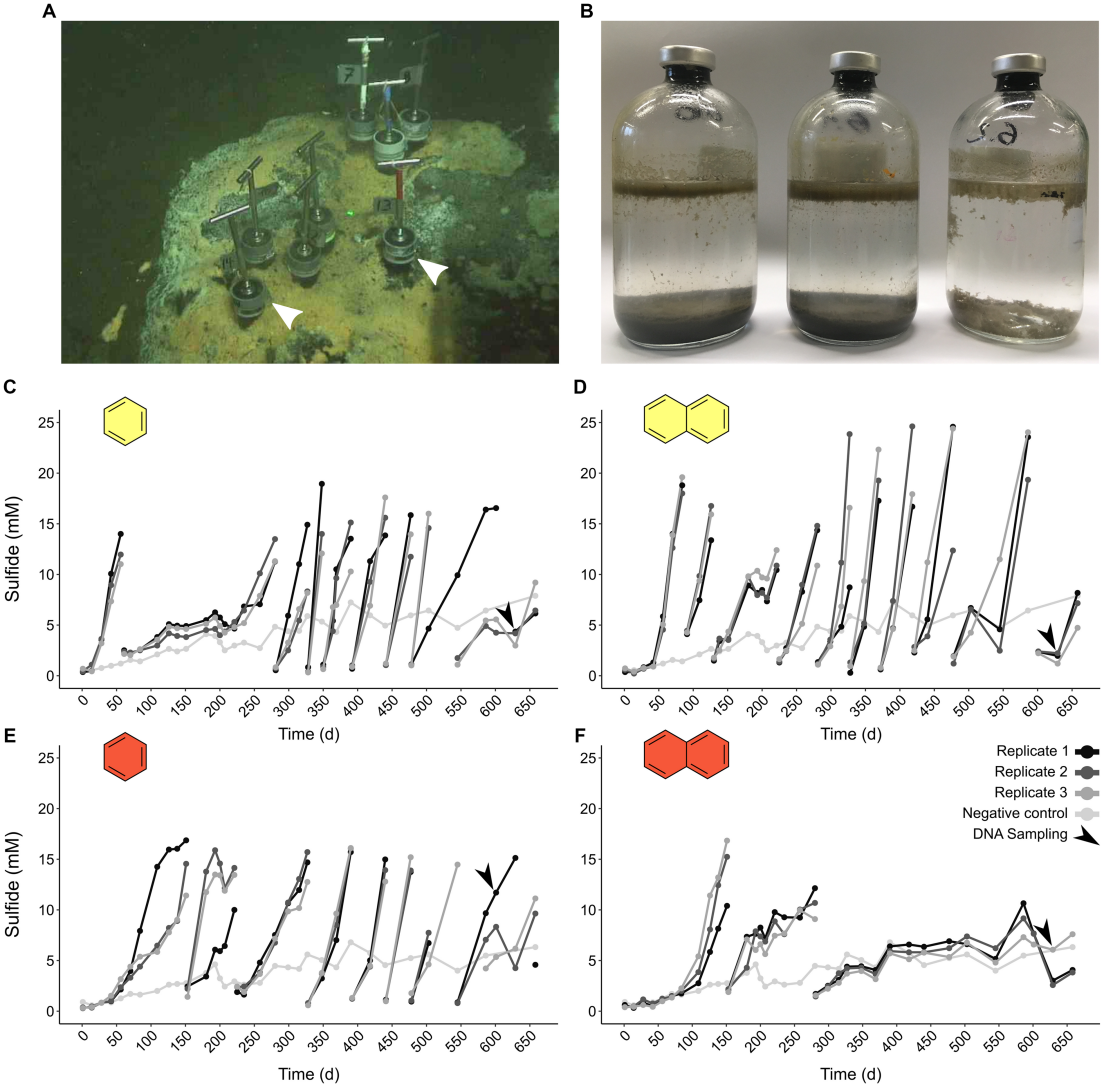
Thermophilic microorganisms from Guaymas Basin sediment oxidize aromatic hydrocarbons. **(A)** Sampling site of petroleum hydrocarbon-rich push cores in the Guaymas Basin. Push cores used for anoxic incubations are indicated by white arrow heads. **(B)** Sequential dilution of sulfide-producing anoxic slurries strongly reduced sediment content (from left to right: original slurry, first dilution, second dilution). **(C–F)** Sulfide production in anoxic cultures supplied with benzene **(C,E)** and naphthalene **(D,F)** incubated at 50°C (yellow filling) and 70°C (red filling). Gaps in sulfide profiles indicate dilution events.

We incubated triplicate batches of an oil-rich sediment slurry from the GB (Figure 1A) with UAHs ranging from one to four aromatic rings at 50°C and 70°C (benzene, naphthalene, phenanthrene, and pyrene, increasing in size). Cultures supplied with phenanthrene and pyrene did not produce more sulfide than a substrate-free control at either incubation temperature, indicating that UAH oxidation did not take place. In contrast, cultures supplied with benzene and naphthalene incubated at 50°C and 70°C started to produce sulfide shortly after the incubation start and first reached sulfide levels >10 mM after 40 (B50 culture) to 120 days (N70). Sequential dilutions strongly reduced the sediment content in the B50, B70, and N50 cultures (Figure 1B). The average doubling time based on sulfide production was 20 days in the N50, 25 days in the B70, and 37 days in the B50 cultures, respectively (Figures 1C–E; Supplementary Table S4). In the N70 cultures, dilutions resulted in decreasing sulfide production rates (Figure 1F) accompanied by long doubling times (>200 days) after the second dilution (Supplementary Table S4).

### Community compositions in the enrichment cultures

After more than 1.5 years of cultivation, we retrieved short-read metagenomes from the original sediment used for incubations and from the four active cultures B50, B70, N50, and N70.

**Figure 2:**
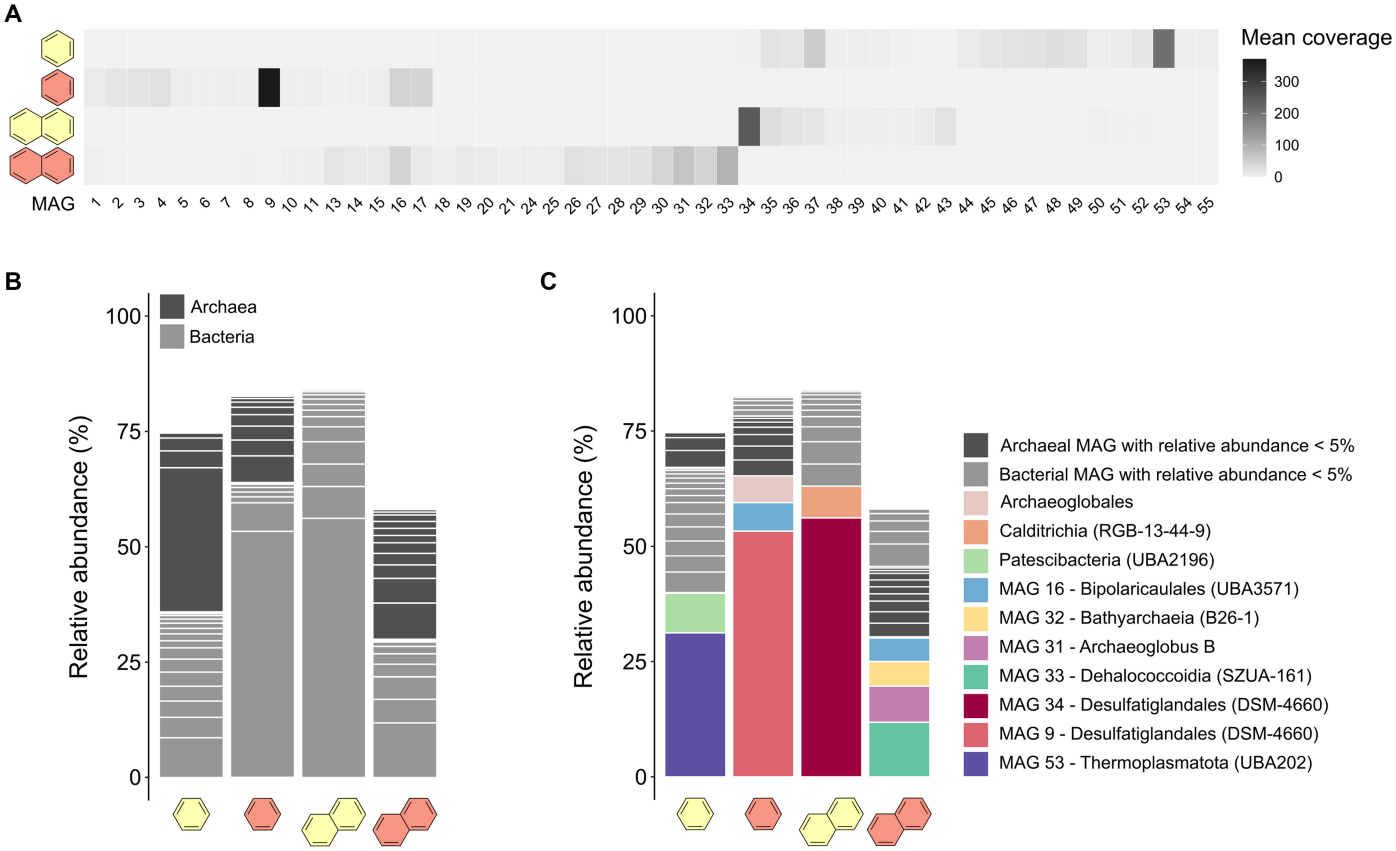
Microbial community compositions differ in anaerobic UAH-degrading cultures depending on substrate and incubation temperature. **(A)** Mean coverages of the 47 MAGs reconstructed from the co-assembly of metagenomes of benzene- and naphthalene-supplied cultures at 50°C and 70°C. **(B)** Relative abundances of MAGs assigned to the domains archaea or bacteria in the four metagenomes. **(C)** Relative abundances of the MAGs on order-level.

From the co-assembled metagenomes, we reconstructed 47 MAGs with completeness >50% and redundancy <10%. Cultures that showed high sulfate-dependent substrate turnover (B50, B70, and N50) were more enriched in specific taxa, whereas the microbial community of the less active N70 culture remained more diverse (Figures 2A,B; Supplementary Table S5). Previous studies observed high archaea:bacteria ratios in heated GB sediments, which increase further with temperature (McKay et al., 2016; Ramírez et al., 2021). In our case, the B50 culture had a higher archaea:bacteria ratio than the B70 culture, contrasting this hypothesis. In the N50 culture, archaea were completely absent, but made up around 50% of the community in the N70 culture, coinciding with literature (Figure 2B; Supplementary Table S5).

In the B50 culture, the most abundant MAG (MAG 53, relative abundance 31%) was classified as species VBQP01 sp008297795 of the archaeal phylum Thermoplasmatota (Figure 2C; Table 1). This MAG belongs to the same species (ANI = 98.9%) as a MAG reconstructed from an environmental metagenome from the GB, M8_bin1702 (GCA_008297795.1). A recent study discussed the potential of this organism to degrade aromatic compound degradation via the phenylacetic acid (PAA) pathway (Liu et al., 2020). The B70 and N50 cultures were dominated by a single bacterial MAG with relative abundance >50%: MAG 9 in the B70 culture and MAG 34 in the N50 culture. Both MAGs were classified as Desulfatiglandales (Table 1). MAG 34 was classified up to species-level and belongs to the genus B111-G9, the representative of which was previously reconstructed from GB sediments (Dombrowski et al., 2018). MAG 9 was only classified at order level. In the N70 culture, MAG 33 affiliated with the bacterial family SpSt-899 of the Dehalococcoidia order SZUA-161 was the most abundant (relative abundance 12%), closely followed by archaeal MAGs of the genus *Archaeoglobus* (MAG 31, 8%), and the family WUQV01 (class Bathyarchaeia; MAG 32, 5.3%), and a bacterial MAG (MAG 16, 5%) of the *Bipolaricaulaceae*. The communities of the cultures differed strongly from the ANME-1 dominated sediment slurry, where the enriched MAGs were only present at very low abundances (0.0–0.1%; Supplementary Table S6).

**Table 1:** MAGs with relative abundance ≥5% recovered from benzene- and naphthalene-oxidizing cultures at 50°C and 70°C.

MAG	Culture	Relative abundance (%)	OGT (°C)	Domain	Phylum	Class	Order	Family	Genus	Species
53	B50	31.2	59.7	Archaea	Thermoplasmatota	E2	UBA202	DSCA01	VBQP01	VBQP01 sp008297795
37	B50	8.6	47.7	Bacteria	Patescibacteria	ABY1	UBA2196	GWA2-42-15		
9	B70	53.3	65.8	Bacteria	Desulfobacterota	DSM-4660	Desulfatiglandales			
16	B70/N70	6.2/5.1	56.0	Bacteria	Bipolaricaulota	Bipolaricaulia	Bipolaricaulales	Bipolaricaulaceae	UBA3571	
17	B70	5.8	78.6	Archaea	Halobacteriota	Archaeoglobi	Archaeoglobales	Archaeoglobaceae	B5-G16	
34	N50	56.2	54.5	Bacteria	Desulfobacterota	DSM-4660	Desulfatiglandales	Desulfatiglandaceae	B111-G9	
35	N50	6.9	47.5	Bacteria	Calditrichota	Calditrichia	RBG-13-44-9	RBG-13-44-9		
33	N70	11.8	60.1	Bacteria	Chloroflexota	Dehalococcoidia	SZUA-161	SpSt-899		
31	N70	7.8	92.1	Archaea	Halobacteriota	Archaeoglobi	Archaeoglobales	Archaeoglobaceae	Archaeoglobus_B	
32	N70	5.3	84.4	Archaea	Thermoproteota	Bathyarchaeia	B26-1	WUQV01		

Optimal growth temperature (OGT) was predicted with OGT_prediction and taxonomic affiliation was determined with GTDB-Tk. B50, benzene-oxidizing culture at 50°C; B70, benzene-oxidizing culture at 70°C; N50, naphthalene-oxidizing culture at 50°C; N70, naphthalene-oxidizing culture at 70°C.

### UAH degradation pathways in abundant MAGs

**Figure 3:**
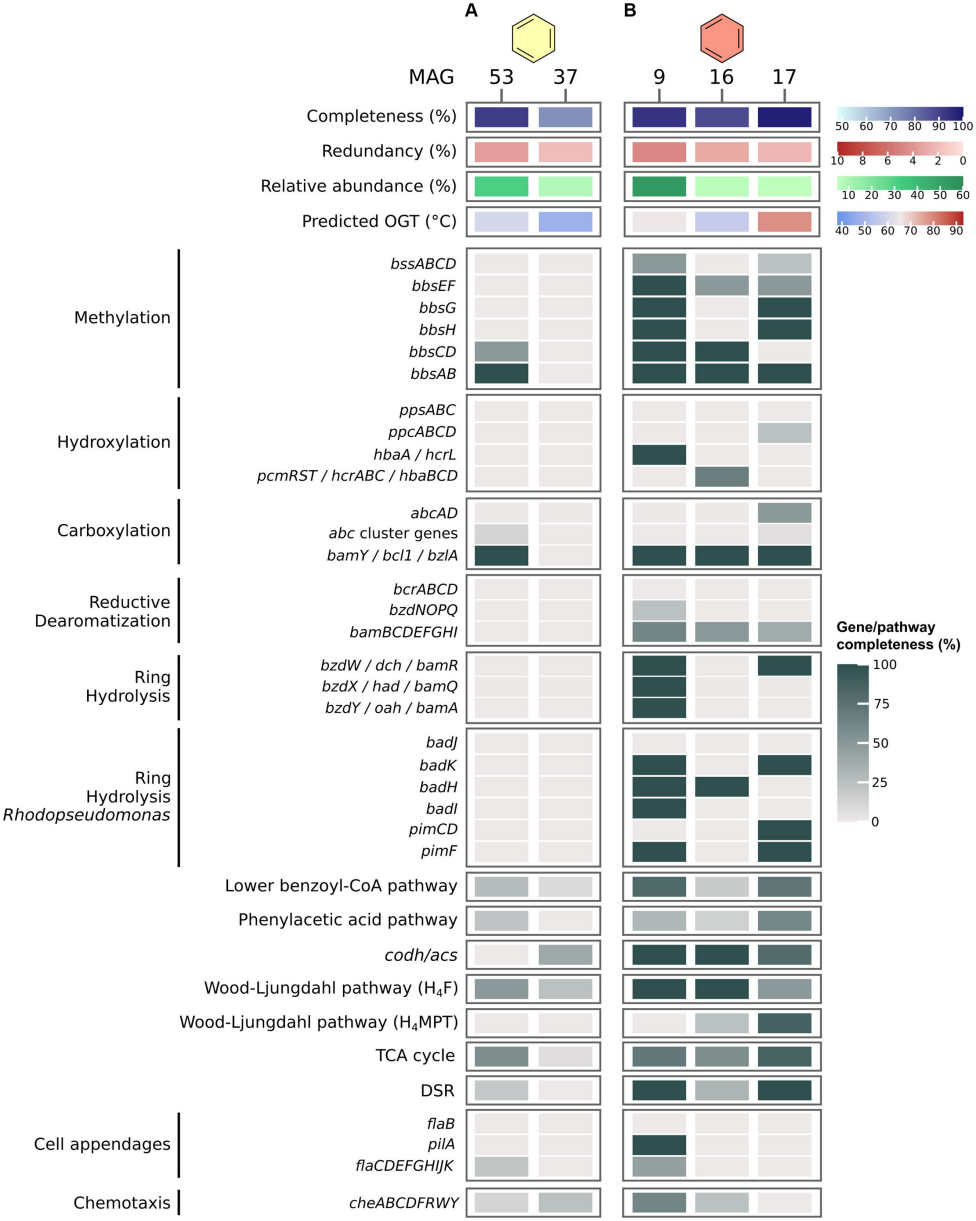
Benzene degradation pathways in MAGs recovered from benzene-oxidizing cultures at 50°C **(A)** and 70°C **(B)**. Genes were identified via BLASTp search against local databases of proteins of interest. For proteins and pathways encoded by several genes, completeness was calculated as percentage of present genes of total genes of the pathway/protein. For pathway genes and abbreviations see Supplementary Table S7.

In the B50 culture, the most abundant MAG, MAG 53 (Thermoplasmatota), encodes only very few proteins of known pathways for the activation and oxidation of benzene, among others benzoylsuccinyl-CoA thiolase (BbsAB) of the methylation pathway, and benzoate-CoA ligase (BamY) of the carboxylation pathway (Figure 3A). We also searched for genes encoding the PAA pathway recently discussed by Liu et al. (2020). MAG 53 encodes only three of thirteen proteins of the pathway, among others the key enzyme phenylacetate-CoA ligase (PaaK; Jiao et al., 2022). The second most abundant MAG, MAG 37 (Patescibacteria), contains even fewer genes for anaerobic benzene oxidation, and is thus a less likely candidate for benzene oxidation. Further, MAG 53 encodes only one (Sat) of three proteins of the DSR pathway. In MAG 37, all genes encoding the DSR pathway are absent. Thus, neither of those two MAGs seems capable of sulfate reduction. We searched for the relevant pathway genes in less abundant MAGs with relative abundances of 3–5% (MAGs 48, 49, 47, 46, and 35; Supplementary Figure S1; Supplementary Table S8). MAGs 46 and 47, affiliated with Desulfatiglandales, encode a majority of the genes for benzene degradation after methylation. In addition, MAG 47 encodes a partial hydroxylation pathway. While neither MAG encodes homologues of Abc, both contain *bamY* and complete or near complete pathways for reductive dearomatization (RD), ring hydrolysis (RH), lower BCoA pathway, CO-Dehydrogenase/Acetyl-CoA Synthase complex (ACDS), and the H_4_F methyl branch of the Wood-Ljungdahl (H_4_F WL) pathway. In addition, both MAGs encode complete DSR pathways. Thus, both organisms could be capable of benzene degradation and can perform DSR. However, because of their low abundance, it is questionable whether they are the main benzene oxidizers in the culture. It is possible that the highly abundant MAG 53 uses a yet unknown mechanism for benzene oxidation. In that case, this archaeon would require partner organisms, potentially the Desulfatiglandales bacteria represented by MAGs 46 and 47, for shuttling electrons into sulfate reduction. This would imply syntrophic interactions within this culture. All three MAGs 46, 47, and 53 encode membrane-bound [NiFe] hydrogenases, which would enable electron exchange via hydrogen. In addition, MAGs 46 and 47 encode archaeal type (FlaB) and bacterial type (PilA) cell appendages (Supplementary Figure S1), and all three MAGs encode multi-heme cytochromes, which could facilitate direct interspecies electron transfer (DIET). In this hypothetical scenario, the role of the MAGs that are more abundant than MAGs 46 and 47, but less abundant than MAG 53, i.e., the MAGs 37, 48, and 49, can be speculated upon. MAG 37 (8.6%) affiliates with the phylum Patescibacteria, previously called Candidate Phyla Radiation (Parks et al., 2018). Based on their small cell and genome size and their limited metabolic potential, symbiotic interactions were previously proposed for members of this phylum (Kantor et al., 2013; Tian et al., 2020; Kuroda et al., 2022). In accordance with this, MAG 37 consists of only ~0.9 Mbp. Some Patescibacteria attach specifically to cells of methanogens of the genus *Methanothrix* in anaerobic wastewater treatment sludge (Kuroda et al., 2022). The infected *Methanothrix* cells exhibited decreased ribosomal activities and physical deformations. Therefore, the authors concluded that the Patescibacteria are likely parasites of the methanogens (Kuroda et al., 2022). Thus, in our culture the Patescibacteria might grow as parasites of the abundant Thermoplasmatota. MAG 48 (4.4%) affiliates with the phylum Caldisericota. This bacterial group was originally referred to as candidate phylum OP5 (Mori et al., 2009). Members of OP5 were present in a methanogenic enrichment culture degrading the monoaromatic compound terephthalate (Lykidis et al., 2011). The authors speculated that the OP5 populations within the culture might remove the final terephthalate oxidation product CO_2_ by shuttling it into butyrate and potentially acetate production (Lykidis et al., 2011). The Caldisericota in our culture may have a similar function. While the H_4_F WL pathway for CO_2_ fixation is only 50% complete in MAG 48, it encodes both phosphate butyryltransferase and butyrate kinase for butyrate synthesis, plus acetate kinase for acetate synthesis. The produced butyrate and/or acetate may then fuel other microbial groups which could oxidize butyrate through the *β*-oxidation pathway and/or convert acetate to acetyl-CoA for various purposes. MAG 49 (3.7%), which also affiliates with Thermoplasmatota, lacks acetate kinase for conversion of acetate to acetyl-CoA, but encodes a *β*-oxidation pathway for utilization of butyrate. In conclusion, multiple symbiotic interactions might co-exist in this culture. More studies are required to elucidate the mechanisms in this complex but highly active culture.

In the B70 culture, MAG 9 (Desulfatiglandales) encodes an almost complete pathway for benzene activation via methylation, and an almost complete class II BCR for RD (Figure 3B). Because *abcAD* is absent from this MAG, and only a single enzyme (HbaA/HcrL) of the hydroxylation pathway is encoded, substrate activation most likely occurs via methylation. MAG 9 encodes a *Thauera* type RH pathway including BamR, BamQ, and BamA, and an almost complete *Rhodopseudomonas* type RH pathway. In addition, MAG 9 encodes a major part of the lower BCoA pathway and CODH/ACS complex, and a complete H_4_F WL pathway for a complete oxidation of benzene to CO_2_. Encoding a complete DSR pathway, this organism is able to combine benzene oxidation with sulfate reduction in a single cell. MAG 9 also encodes type IV pilin (PilA) and several chemotaxis genes, which could enable it to outcompete other potential benzene degraders and increase its efficiency for benzene degradation, as previously shown for the naphthalene-degrading bacterium *Pseudomonas putida* G7 (Law and Aitken, 2003). The estimated OGT of 65°C for this MAG supports the thermophilic character of this organism. Low-abundance MAGs in this culture, like MAG 16 (Bipolaricaulales) and MAG 17 (Archaeoglobales), also encode proteins for anaerobic benzene degradation, such as BamY, some subunits of class II BCR, and in case of MAG 17 a complete DSR pathway. Notably, MAG 17 encodes one of two subunits of anaerobic benzene carboxylase (AbcA), which could enable it to degrade benzene via carboxylation. It is thus possible that the two organisms contribute to a small degree to benzene oxidation and/or sulfate reduction, but considering abundances, we expect the organism represented by MAG 9 to be the main active organism in the culture.

**Figure 4:**
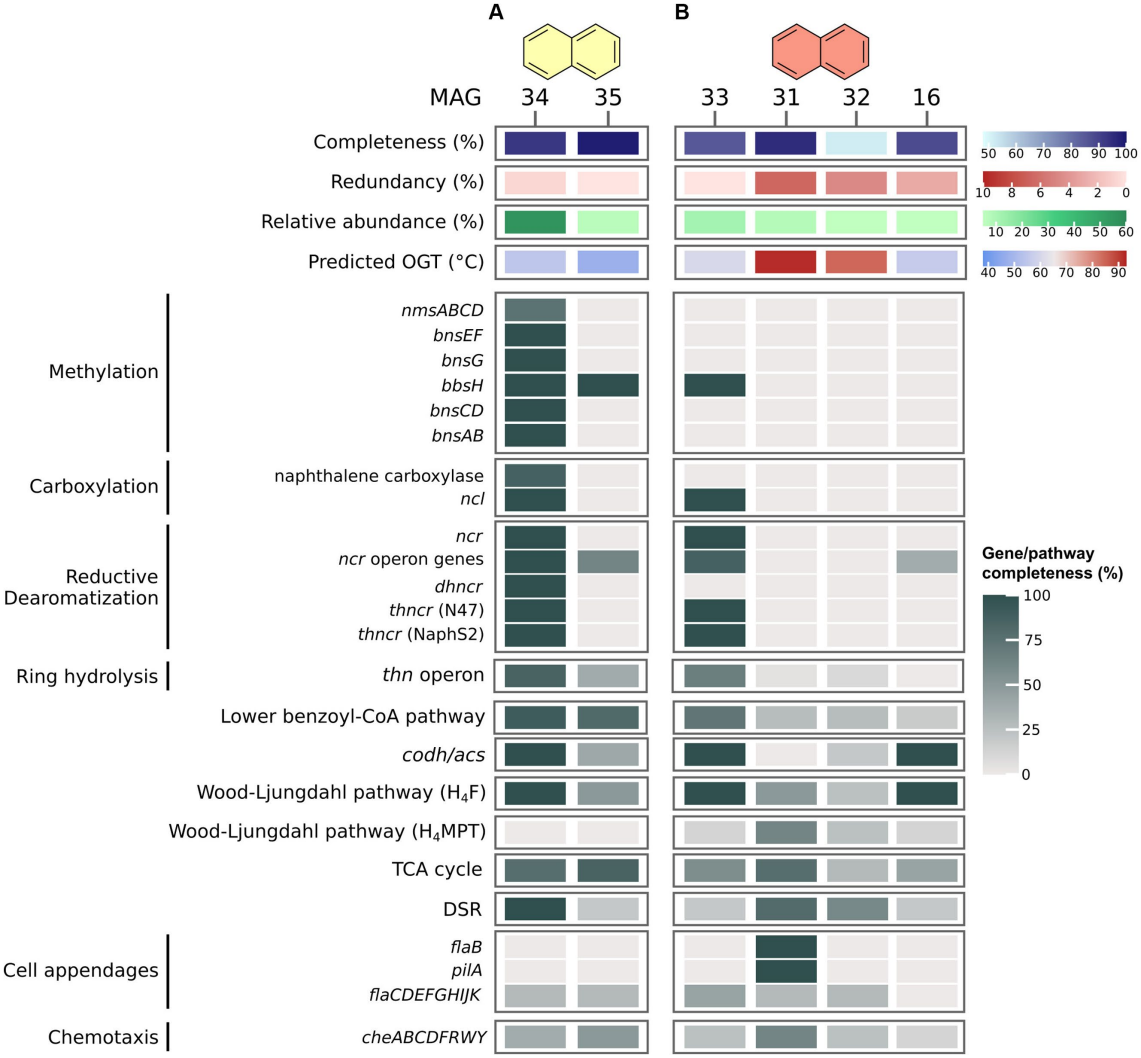
Naphthalene degradation pathways in MAGs recovered from naphthalene-oxidizing cultures at 50°C **(A)** and 70°C **(B)**. Genes were identified via BLASTp search against local databases of proteins of interest. For proteins and pathways encoded by several genes, completeness was calculated as percentage of present genes of total genes of the pathway/protein. For pathway genes and abbreviations see Supplementary Table S7.

In the N50 culture (Figure 4A), MAG 34 (Desulfatiglandales) exhibits vast genomic capacities for anaerobic naphthalene oxidation. First, it encodes an almost complete pathway of the known enzymes of the methylation pathway. It is also capable of activation via direct carboxylation, encoding homologues of all eight genes of the naphthalene carboxylase complex. This complex consists of three UbiD-like carboxylases, two ParA-MiND ATPase-like-proteins, and three putative linker proteins (Koelschbach et al., 2019). Moreover, MAG 34 encodes four copies of naphthoyl-CoA ligase (NCL) highly homologous to the variants of NaphS2 and N47 (amino acid identity ≥67%). NCL converts 2-naphthoate to 2-naphthoyl-CoA (Heker et al., 2023). Further, it is capable of the three-step RD of naphthalene, encoding homologues of 2-naphtoyl-CoA reductase (NCR), 5,6-dihydro-2-naphthoyl-CoA reductase (DHNCR), and both N47 and NaphS2 type of 5,6,7,8-tetrahydro-2-naphthoyl-CoA reductase (THNCR). An almost complete *thn* operon and lower BCoA pathway facilitates RH and oxidation to acetyl-CoA, followed by complete oxidation to CO_2_ via the CODH/ACS complex and the remaining H_4_F WL pathway. MAG 34 encodes a complete DSR pathway, enabling the organism to shuttle electrons from naphthalene oxidation directly into sulfate reduction. The incubation temperature is very close to the estimated OGT of the organism of 55°C. Its high relative abundance and its extensive genomic capacity for naphthalene degradation suggest that the bacterium represented by MAG 34 is the dominant, maybe even the only naphthalene oxidizer in the culture.

In the N70 culture (Figure 4B), MAG 33 (Dehalococcoidia) encodes only one protein of the methylation pathway, and lacks naphthalene carboxylase, thus limiting its options for naphthalene activation. Yet, MAG 33 encodes NCL, two of the three reductases required for RD, and about two thirds of the *thn* operon for RH, among others the putative ring-cleaving hydrolase ThnL (Meckenstock et al., 2016). Further, it encodes an almost complete lower BCoA pathway, plus a complete CODH/ACS and H_4_F WL pathway, which would enable it to oxidize naphthalene to CO_2_. MAG 33 is about 85% complete, thus it is possible that the missing 15% encode naphthalene-activating UbiD-like carboxylases and DHNCR, which would enable it to degrade naphthalene. Because none of the other MAGs with relative abundances ≥5% in this culture encode numerous genes for activation, RD, or RH, MAG 33 is the most likely candidate for naphthalene oxidation. MAG 33 encodes only one of three proteins of the DSR pathway, sulfate adenylyltransferase (Sat), and is therefore probably incapable of sulfate reduction. The next most abundant MAG, MAG 31 (Archaeoglobales), encodes an almost complete DSR pathway, which lacks only the alpha subunit of Apr. Thus, it is possible that naphthalene oxidation in this culture occurs via syntrophic interactions. While membrane-bound hydrogenases are absent in MAG 33, both MAGs 33 and 31 encode formate dehydrogenases, which could facilitate electron transfer from MAG 33 to MAG 31 via formate. Direct transfer via DIET is also conceivable, because MAG 31 encodes both FlaB and PilA, and both MAG 33 and MAG 31 encode several multi-heme cytochromes.

We aimed to bring the results from our enrichment cultures into a wider ecological context and examined the distribution of AH degradation genes and pathways in the larger taxonomic groups of the microorganisms that we enriched in our study. We refrained from examining the phylogeny of the Thermoplasmatota MAG 53 (M8_bin1702), because the Thermoplasmatota phylogeny was well-resolved in the recent study by Liu et al. (2020). Thus, we focused on two bacterial groups with which abundant MAGs from our cultures were affiliated: the order Desulfatiglandales (class DSM-4660) and the order SZUA-161 (class Dehalococcoidia).

### Environmental distribution of Desulfatiglandales and SZUA-161

**Figure 5:**
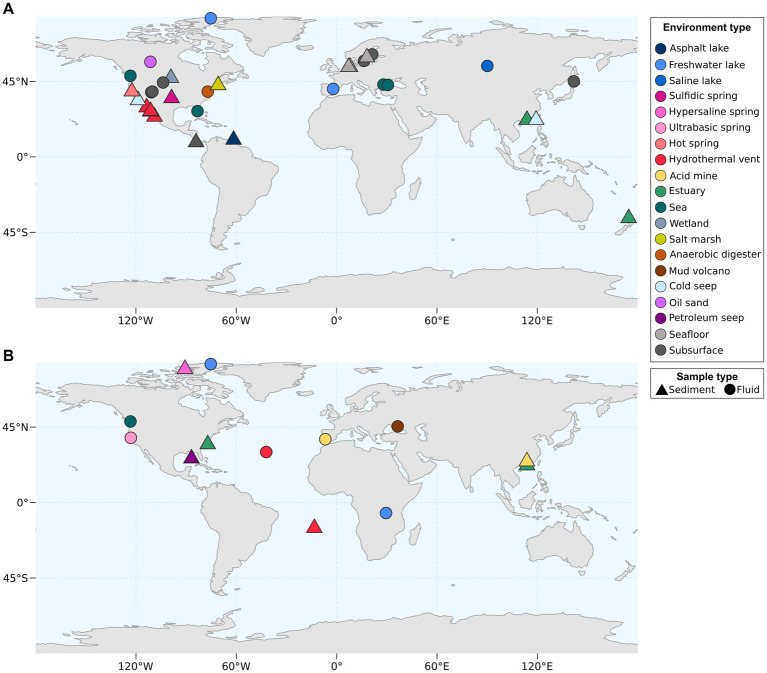
Environmental origin of bacterial MAGs of the Desulfobacterota order Desulfatiglandales **(A)** and of the Dehalococcoidia order SZUA-161 **(B)**. Coordinates were acquired from metadata accompanying the MAGs deposited at NCBI.

Desulfatiglandales currently comprise 135 publicly available MAGs on GDTB, while for SZUA-161 only 26 MAGs are available at the moment. Both Desulfatiglandales and Dehalococcoidia are globally widespread members of marine sediment and subsurface communities (Inagaki et al., 2006; Parkes et al., 2014; Wasmund et al., 2014; Robador et al., 2016). Desulfatiglandales MAGs have been recovered mainly from or near the North American continent. Fewer MAGs originate from Eurasia, including the Black Sea, with only one MAG (GCA_024641835.1) stemming from the Southern Hemisphere (Figure 5A). The few available SZUA-161 MAGs have been reconstructed from continental samples from North America, Eurasia, Asia, and Africa, plus from marine samples from the Atlantic (Figure 5B). Both group distributions likely reflect sampling efforts rather than actual occurrence and/or abundance.

The MAGs of both orders originate from a wide array of environments, including lakes, springs, cold seeps, the seafloor and the subsurface. More than half of publicly available Desulfatiglandales MAGs (75 out of 135) originate from hydrothermal vent sediment, mainly from the GB (70 MAGs) and to a smaller degree (5 MAGs) from the Pescadero Basin, a recently described hydrothermal vent area (Paduan et al., 2018) located ~400 km southeast of the GB in the Gulf of California. Further MAGs include 17 MAGs from groundwater or aquifer samples, 9 MAGs from seawater samples from the Black Sea, the Pacific coast, and the Gulf of Mexico, and 7 MAGs from estuary sediment. For SZUA-161, hydrothermal vents are also the most common environment type, with 11 MAGs emanating from the Lost City hydrothermal field located at the Mid-Atlantic Ridge (Kelley et al., 2001). Considering the current data on both bacterial groups, it seems plausible that both are widely distributed and capable of inhabiting diverse environments, with a potential preference for hydrothermal vent areas.

Next, we examined the phylogenomic placement of Desulfatiglandales MAGs and the SZUA-161 MAG from our study in their larger taxonomic groups, and investigated the genomic potential of environmental Desulfatiglandales and SZUA-161 MAGs for anaerobic UAH degradation.

### Genomic capacity for UAH degradation in Desulfatiglandales bacteria

According to GTDB (Parks et al., 2022), Desulfatiglandales is currently the only order in the class DSM-4660, which in turn belongs to the phylum Desulfobacterota. Desulfatiglandales are defined as gram-negative, rod-shaped, strictly anaerobic, and mesophilic bacteria that utilize AH derivatives like phenol and benzoate as electron donors in combination with the reduction of sulfate and other inorganic sulfur compounds (Suzuki et al., 2014; Waite et al., 2020; Galushko and Kuever, 2021). Notable members of the order include NaphS2 (Galushko et al., 1999; DiDonato et al., 2010), *Desulfatiglans anilini*, which degrades phenol and the aromatic amine aniline (Schnell et al., 1989; Ahn et al., 2009; Suzuki et al., 2014), and the recently enriched phenanthrene-degrader candidate *Desulfatiglans* TRIP_1 (Himmelberg et al., 2018; Kraiselburd et al., 2019).

**Figure 6:**
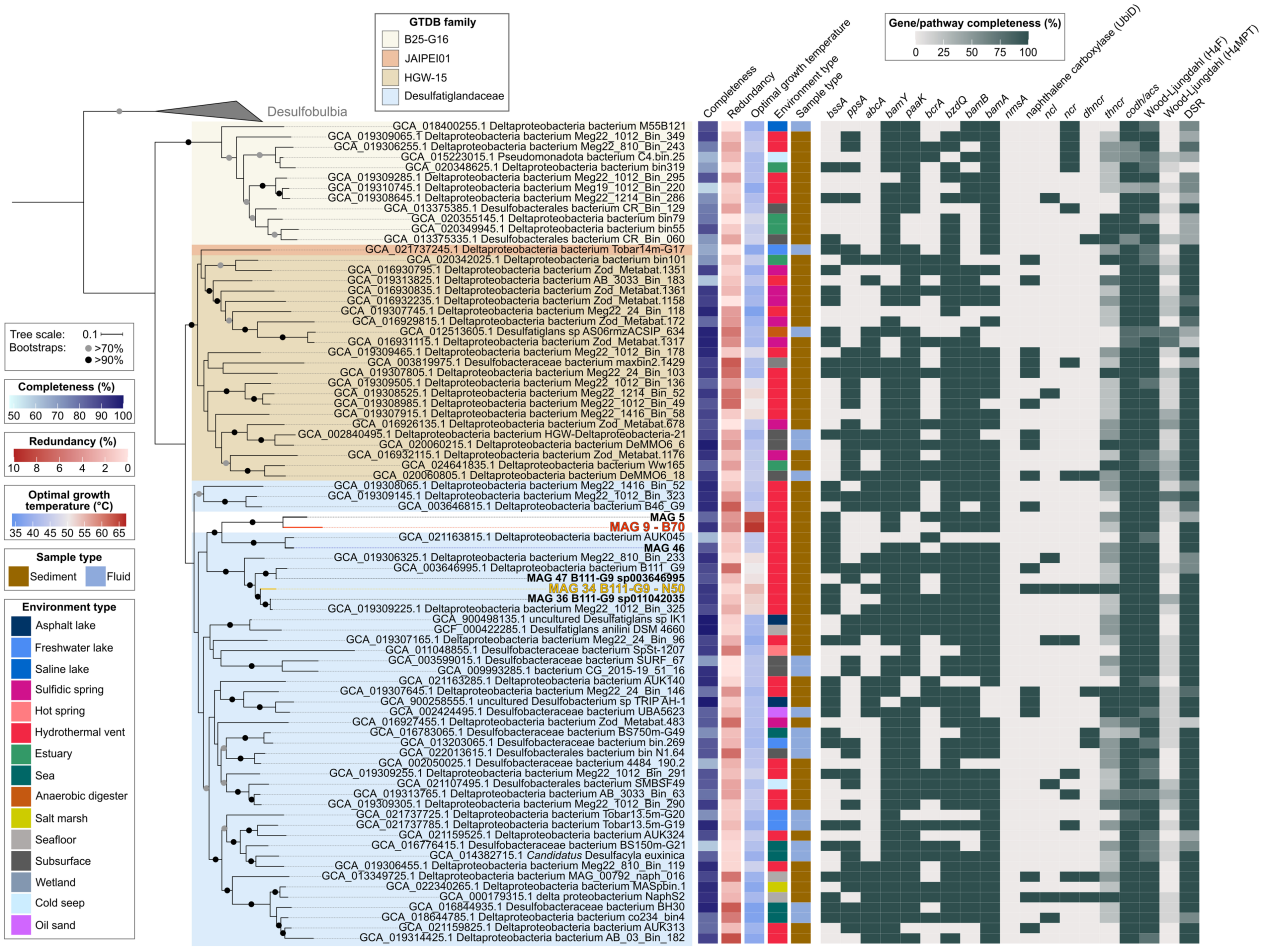
Genomic capacities for anaerobic aromatic hydrocarbon degradation in genomes of the bacterial order Desulfatiglandales. MAGs recovered in this study are highlighted in bold, MAG 9 dominant in the benzene 70°C (B70) culture and MAG 34 dominant in the naphthalene 50°C (N50) culture are additionally highlighted in red and yellow, respectively. For pathway genes and abbreviations see Supplementary Table S7.

Desulfatiglandales include four families: *Desulfatiglandaceae* with the type genus *Desulfatiglans* (Waite et al., 2020), B25-G16, HGW-15, and JAIPEI01. The majority (77) of publicly available MAGs fall into the family *Desulfatiglandaceae*, with 34 MAGs classified as HGW-15, 26 MAGs as B25-G16, and only one MAG representing JAIPEI01. This phylogeny is well-resolved in our phylogenomic tree based on concatenated SCGs (Figure 6). The tree is divided into two large monophyletic groups: (1) the B25-G16 family and (2) the three other families JAIPEI01, HGW-15, and *Desulfatiglandaceae*. All six MAGs from our study fall into one monophyletic clade within the family *Desulfatiglandaceae*, which indicates that MAGs 5 and 9 are part of this family, even though they were not assigned as such by GTDB-Tk. This clade contains four additional MAGs (GCA_021163815.1, GCA_019306325.1, GCA_003646995.1, and GCA_019309225.1), all from hydrothermal vent sediment in the GB and the Pescadero Basin. The clade splits into two groups: (1) MAGs 5, 9, and 46 and GCA_021163815.1 (2) GCA_019306325.1, GCA_003646995.1, MAGs 47, 34, 36, and GCA_019309225.1. Based on ANI, AAI and the tree structure (Figure 6; Supplementary Figures S2, S3; Supplementary Table S9), group one consists of two genera, one containing two species, represented each by MAGs 5 and 9 (ANI 78%, AAI 73%), and one containing one species represented by two MAGs, GCA_021163815.1 and MAG 46 (ANI 95%, AAI 96%; Konstantinidis et al., 2017; Jain et al., 2018). Notably, MAGs 5 and 9 exhibit the highest estimated OGTs of all Desulfatiglandales MAGs (63°C and 66°C, respectively compared to an average OGT of 44°C of all publicly available MAGs; Supplementary Table S1). Therewith, we were able to reconstruct the MAGs of the, to this date, likely most thermophilic genus of the class, and enrich the currently most thermophilic organism and anaerobic AH degrader of the clade, represented by MAG 9, at temperatures slightly above its predicted OGT. Strikingly, the MAGs of the sister genus, MAG 46 and GCA_021163815.1, likely grow at mesophilic temperatures about 20°C lower (OGT 44°C). According to the same criteria, the second group consists of four species of one genus: species (1) GCA_019306325.1; species (2) GCA_003646995.1 and MAG 47; species (3) MAG 34; and species (4) MAG 36 and GCA_019309225.1. Members of group two are predicted moderate thermophiles with OGTs around 50–55°C, which coincides with the enrichment of the MAG 34 bacterium at 50°C.

Genes coding for central enzymes for the anaerobic AH metabolism, e.g., *bamY* and *bamA*, are widely distributed within the order Desulfatiglandales, and do not appear to be connected to specific clades. Most MAGs also encode PaaK, the key enzyme of the PAA pathway. CODH/ACS and bacterial-type H_4_F WL pathway are also ubiquitously present and should allow a downstream oxidation of aromatic compounds. The DSR pathway is strongly represented for coupling to sulfate reduction, even though the pathway is incomplete in about a third of the included MAGs. In some cases this may be a result of low completion, e.g., in MAGs GCA_015223015.1 and GCA_016776415.1.

About half of the MAGs encode the alpha subunit of benzylsuccinate synthase (BssA) of the methylation pathway of benzene and the alpha subunit of phenylphosphate synthase (PpsA) of the hydroxylation pathway. Interestingly, more than a third of the Desulfatiglandales MAGs encode AbcA. AbcA, in connection with BamY, could enable many yet uncultured Desulfatiglandales of degrading benzene via the carboxylation pathway. Regarding BCRs, the *bcr* type BCR I, isolated from *Thauera aromatica* and *Rhododpseudomonas palustris* (Song and Ward, 2005), is the least distributed version, with about a third of MAGs encoding the catalytic subunit BcrA. More than three quarters of MAGs encode BzdQ, the active subunit of *bzd* type BCR I of *Azoarcus evansii* (Song and Ward, 2005). The BamB subunit of ATP-independent class II BCR is similarly as represented as BzdQ, thus both ATP-dependent and -independent BCRs seem to be used by Desulfatiglandales.

Genes for the anaerobic activation of naphthalene are less frequent in Desulfatiglandales than genes for benzene activation. We did not detect genes encoding the alpha subunit of naphthyl-2-methylsuccinate synthase (*nmsA*) for naphthalene degradation after methylation (Selesi et al., 2010), in any of the MAGs. Instead, about a quarter of MAGs encode one or more copies of the UbiD-carboxylases previously identified in the naphthalene carboxylase operon (Koelschbach et al., 2019). NCL-encoding genes are present in only eight MAGs, NCR-encoding genes in 15 MAGs, DHNCR-encoding genes in 8 MAGs, and the complete operon encoding THNCR in 12 MAGs. The combined presence of all genes required for naphthalene degradation via carboxylation is rare. In fact, next to the known naphthalene-degrader NaphS2, MAG 34 from the N50 culture is the only MAG encoding the complete naphthalene degradation pathway. MAG 34 also contains key genes for the anaerobic activation of benzene, *bssA* and *abcA*. Thus this bacterium might also be capable of benzene and/or benzene derivate degradation.

### Genomic capacity for UAH degradation in SZUA-161 bacteria

In GDTB, the order SZUA-161 falls into the class Dehalococcoidia and phylum Chloroflexota (Parks et al., 2022). While this specific order is not well-described yet, cultured Dehalococcoidia are known for the reductive dehalogenization of chlorinated and brominated compounds (Maymó-Gatell et al., 1997; Yan et al., 2009). Examples include *Dehalococcoides mccartyi*, which reduces chlorinated ethenes and chlorinated benzenes (Löffler et al., 2013), and the *Dehalogenimonas* species *D. lykanthroporepellens* and *D. alkenigignens*, which reduce poylchlorinated aliphatic alkanes (Moe et al., 2009; Bowman et al., 2013). Both genera fall into the order Dehalococcoidales and use hydrogen as electron donor. Recent studies suggest a potential for both aerobic and anaerobic aromatics degradation in the Dehalococcoidia. For instance, the phenol derivatives vanillin and syringic acid stimulated growth of aerobic bacteria of the Dehalococcoidia order Tepidiformales isolated from geothermal springs and thriving at 55–60°C (Palmer et al., 2023). Further, Dehalococcoidia MAGs, one of which is part of the order SZUA-161 (GCA_004376205.1), from petroleum seeps in the Gulf of Mexico contained proteins for the anaerobic hydroxylation of the alkylbenzene *p*-cymene and class I BCRs for RD (Dong et al., 2019). Another study enriched a Dehalococcoidia closely related to *D. alkenigignens* anaerobically on lignin, which encoded an almost complete benzoate degradation pathway (Yu et al., 2023). The study further showed that most Dehalococcoidia MAGs recovered from marine sediment encoded *bcr*-type BCRs, which were absent in Dehalococcoidia MAGs from groundwater or seawater.

**Figure 7:**
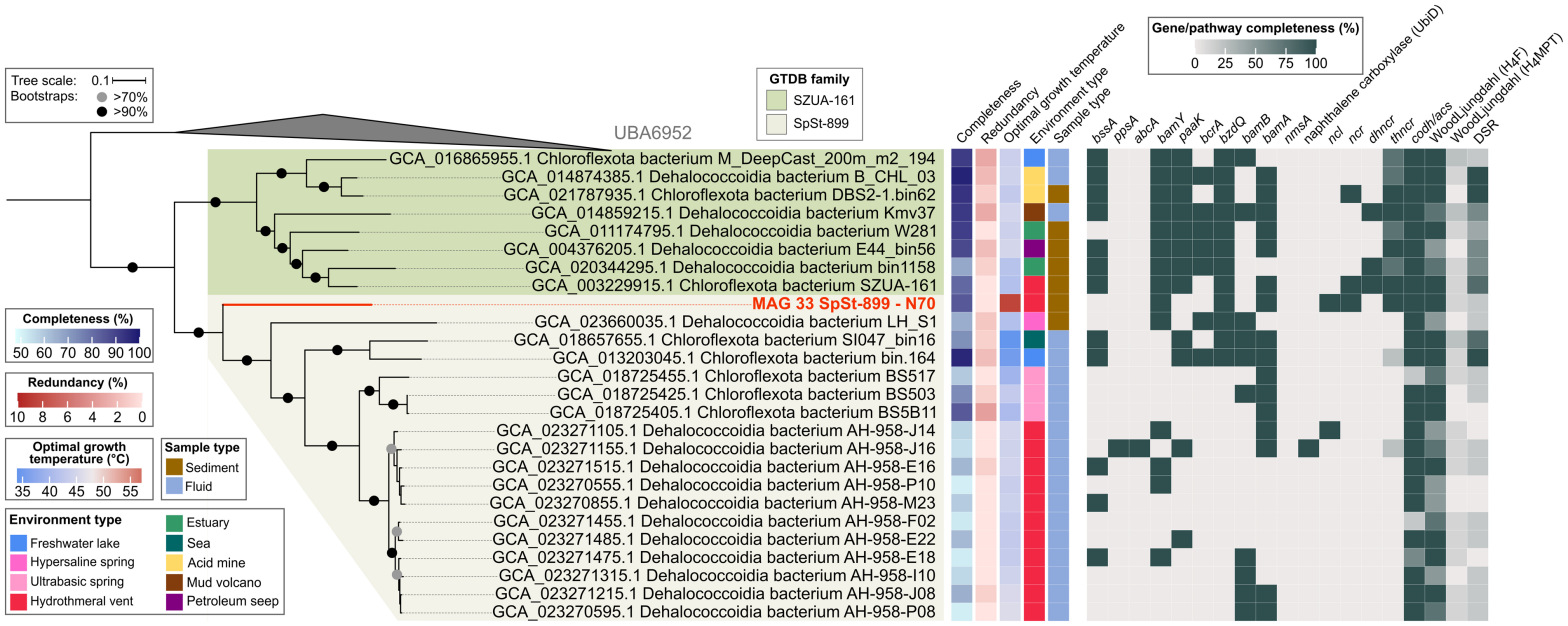
Genomic capacities for anaerobic aromatic hydrocarbon degradation in MAGs of the Dehalococcoidia order SZUA-161. The MAG recovered in this study from the naphthalene 70°C (N70) culture is highlighted in red. For pathway genes and abbreviations see Supplementary Table S7.

The order SZUA-161 currently contains 26 MAGs of two families, SZUA-161 (9 MAGs) and SpSt-899 (17 MAGs). This phylogeny is well-resolved in our tree (Figure 7). MAG 33 from the N70 culture is situated at the root of the SpSt-899 family branch, which coincides with its taxonomic affiliation to this family. Notably, MAG 33 has a much higher estimated OGT (60°C) than all other MAGs of the order (average OGT 43°C). We therefore propose that SpSt-899 was originally more thermophilic, and later distributed into less heated environments. The closest relative to MAG 33 is the Dehalococcoidia bacterium LH_S1 (GCA_023660035.1), which belongs to a different genus (AAI 59%; Supplementary Figures S4A,B; Supplementary Table S10).

The SZUA-161 family encodes several key pathway genes for benzene degradation. For instance, seven out of eight MAGs encode BssA, all MAGs encode BamY, and six MAGs encode BamA. Interestingly, SZUA-161 seem to rely on ATP-dependent BCRs, since all MAGs encode BzdQ, five MAGs encode BcrA, but only two MAGs encode BamB. Naphthalene activation genes, both for the methylation and carboxylation pathway, are not encoded by members of the SZUA-161 family. Yet, two MAGs encode NCR, three MAGs encode DHNCR, and all MAGs encode one or more subunits of the four-subunit THNCR. Thus, while SZUA-161 likely cannot activate naphthalene directly, they might be able to dearomatize naphthyl-derivatives. All SZUA-161 family MAGs encode complete CODH/ACS complexes and partial or complete H_4_F WL pathways, which they could use for oxidation of dearomatized naphthyl-residues to CO_2_. Surprisingly, two MAGs of the SZUA-161 family encode complete DSR pathways, and the other six MAGs encode partial DSR pathways, insinuating that these organisms are capable of sulfate reduction, a metabolic trait that was previously not associated with members of the Dehalococcoidia.

In the SpSt-899 family, four members that are closer to the root of the clade, MAG 33, LH_S1 (GCA_023660035.1), GCA_018657655.1, and GCA_013203045.1, contain several genes for the anaerobic degradation of benzene or derivatives, e.g., *bssA* and *bamY* in two of the MAGs, and *bamA* in three of the MAGs. Those MAGs also encode several *bcrA*, *bzdQ*, and *bamB* subunits of BCRs, indicating a capacity for RD. MAG 33 is the only MAG of the group encoding a partial pathway for naphthalene degradation (NCL, NCR, and THNCR). Interestingly, one MAG of this group, GCA_013203045.1, encodes a complete and another MAG, GCA_018657655.1, an almost complete DSR pathway, indicating capacity for DSR in this group. In the remaining members of SpSt-899, genes for both benzene and naphthalene degradation are scarce. Yet, several MAGs contain isolated genes for the degradation of benzene, i.e., *bssA*, *bamY*, *bamB*, and *bamA*. Curiously, one MAG (GCA_023271155.1) encodes *abcA* for direct carboxylation of benzene and a copy of UbiD-like naphthalene carboxylase, even though it lacks many genes of the downstream degradation pathway. Most SpSt-899 MAGs encode CODH/ACS and the H_4_F WL pathway, even though both components are incomplete in some MAGs. All in all, we propose that capacity for UAH/AH degradation is an ancestral trait of the SpSt-899 and was lost in more recent members. However, particularly in several closely related MAGs of the AH-958 group, to which GCA_023271155.1 also belongs, the analysis is impaired by low completeness (52–66%) of the MAGs (Supplementary Figure S4A; Supplementary Tables S2, S10). More high-quality MAGs are needed for more reliable predictions about UAH/AH degradation capacity in this family.

## Discussion

Most previously established UAH-degrading cultures grow at mesophilic temperatures around 30°C (Galushko et al., 1999; Meckenstock et al., 2000; Musat et al., 2009; Zhang et al., 2012; Dong et al., 2017). A noteworthy exception is the benzene-degrading iron-reducing archaeon *Ferroglobus placidus*, which thrives at 85°C (Holmes et al., 2011). In this study, we aimed to enrich thermophilic UAH-degrading microorganisms in connection to the reduction of sulfate, one of the most abundant terminal electron acceptors in anoxic marine sediments, from GB sediment (Thamdrup, 2000; Jørgensen and Kasten, 2006; Bowles et al., 2014). Previous cultivation efforts using GB sediment revealed UAH oxidation in mesophilic aerobic bacteria degrading naphthalene and phenanthrene (Bazylinski et al., 1989; Goetz and Jannasch, 1993), and anaerobic degradation of benzene by *Desulfatiglans* strains SB-21 and SB-30 at 28–30°C (Phelps et al., 1998). Cultures of anaerobic thermophilic UAH-degraders have, to the best of our knowledge, not previously been established from GB sediment.

In this study, we established benzene- and naphthalene-degrading cultures at 50°C and 70°C. We found distinct communities in each culture, suggesting that sediments contain a large variety of specified archaea and bacteria that can be enriched with different substrates and temperature combinations. Surprisingly, we found only few benzene degradation genes in the highly abundant Thermoplasmatota MAG in the B50 culture. This pathway has been previously detected mostly in bacteria. It is possible that archaeal homologues differ so much from the bacterial enzymes that they could not be detected due to the high stringency of the BLASTp search. The only known archaeal UAH degrader, *F. placidus*, uses a bacterial-type pathway for benzene degradation (Holmes et al., 2011). Alternatively, this Thermoplasmatota archaeon might employ a different, yet unknown mechanism. Recently, it was proposed that Thermoplasmatota archaea from the GB are able to degrade aromatics via the PAA pathway (Liu et al., 2020). Our MAG did include the key gene, *paaK*, but lacked most other genes of this pathway. Plus, this pathway also converges in BCoA, and BCR and the enzymes of the lower BCoA pathway are required for further oxidation, most of which are absent in MAG 53. Yet, the high relative abundance of this MAG suggests an important role in the culture. Whether and by which mechanism this archaeon degrades benzene requires further investigation.

Desulfatiglandales MAGs 9 and 34 were highly abundant in the B70 and N50 cultures, respectively, and encode plenty of genes for anaerobic UAH oxidation and a complete DSR pathway. Thus, they are most likely the UAH oxidizers in their respective cultures and combine UAH oxidation with sulfate reduction in a single cell. MAG 9 lacks AbcAD, the enzyme for direct carboxylation of benzene, which is currently the only confirmed activation mechanism (Abu Laban et al., 2010; Luo et al., 2014; Eziuzor et al., 2022). Instead, it encodes genes for degrading benzene after methylation by a yet unknown enzyme. Further studies are needed to identify the enzyme responsible for the challenging direct methylation of benzene. Yet, our study indicates that carboxylation is not the only pathway used for benzene activation in anoxic sediments. MAG 34 encodes an almost complete operon for the anaerobic oxidation of naphthalene via carboxylation (Koelschbach et al., 2019), and further enzymes for complete naphthalene oxidation. According to our analysis of naphthalene degradation genes in the order Desulfatiglandales, MAG 34 is only the second bacterium in the clade, together with NaphS2, capable of oxidizing naphthalene via direct carboxylation. Grown at 50°C, it is also the most thermophilic anaerobic naphthalene-degrader to date, to the best of our knowledge. With the enrichment of thermophilic species, especially MAG 9 thriving at 70°C, the general definition of Desulfatiglandales as mesophilic may be questioned (Galushko and Kuever, 2021). We did not detect genes for processing of naphthalene (*nmsA*) after activation via direct methylation in the Desulfatiglandales. Thus, methylation does not seem to be a frequent mechanism for naphthalene activation, which is in accordance with previous studies (Zhang and Young, 1997; Musat et al., 2009; DiDonato et al., 2010).

In the N70 culture, we identified MAG 33 of the Dehalococcoidia order SZUA-161 as the most likely naphthalene oxidizer. The genomic potential for anaerobic aromatics degradation was previously reported for the traditionally hydrogenotrophic organohalide-respiring Dehalococcoidia (Calvo-Martin et al., 2022; Palmer et al., 2023). We found such potential also in the order SZUA-161, particularly in the family SZUA-161, as evidenced by the presence of key genes. While a true confirmation of AH/UAH degradation activity of Dehalococcoidia requires further experimental evidence, e.g., transcriptomics and proteomics, it seems that this clade holds more versatile metabolisms than previously believed. Whether Dehalococcidia may be able to combine AH/UAH degradation to dehalogenation is an intriguing question for the future.

Because the GB exhibits similar characteristics to deeply-buried petroleum reservoirs, i.e., high temperatures, absence of oxygen and presence of UAHs in surface-near sediment layers, the microorganisms enriched in this study might thrive in such yet under-sampled reservoirs (Sierra-Garcia and Oliveira, 2013), where they could contribute to reservoir souring (Tanji et al., 2014). Particularly the Desulfatiglandales bacterium in the B70 culture operates close to the temperature limit of reservoir sterilization of 80–90°C (Wilhelms et al., 2001). Future studies using advanced sampling and sequencing techniques could reveal the presence and activity of these bacteria in such reservoirs.

## Data availability statement

The datasets presented in this study can be found in online repositories. The names of the repository/repositories and accession number(s) can be found at: BioProject, PRJNA1013425.

## Author contributions

HZ: Conceptualization, Data curation, Investigation, Methodology, Project administration, Software, Visualization, Writing – original draft, Writing – review & editing, Supervision. CO: Investigation, Methodology, Software, Writing – review & editing. DBM: Investigation, Methodology, Software, Writing – review & editing. GW: Conceptualization, Funding acquisition, Project administration, Resources, Supervision, Writing – review & editing.

## Funding

The author(s) declare financial support was received for the research, authorship, and/or publication of this article. This study was funded by the Deutsche Forschungsgemeinschaft (DFG) through the Cluster of Excellence EXC 2077 “The Ocean Floor—Earth’s Uncharted Interface” (project no. 390741603) and the Max Planck Society (MPS).

## Conflict of interest

The authors declare that the research was conducted in the absence of any commercial or financial relationships that could be construed as a potential conflict of interest.

## Publisher’s note

All claims expressed in this article are solely those of the authors and do not necessarily represent those of their affiliated organizations, or those of the publisher, the editors and the reviewers. Any product that may be evaluated in this article, or claim that may be made by its manufacturer, is not guaranteed or endorsed by the publisher.

## Footnotes

^1^ https://sourceforge.net/projects/bbmap/

^2^ https://github.com/HRGV/phyloFlash

^3^ https://github.com/ablab/spades

^4^ https://github.com/merenlab/anvi’o/releases

^5^ http://bowtie-bio.sourceforge.net/bowtie2/index.shtml

^6^ http://samtools.sourceforge.net

^7^ https://github.com/hyattpd/Prodigal

^8^ https://ccb.jhu.edu/software/centrifuge

^9^ https://ecogenomics.github.io/CheckM

^10^ https://github.com/Ecogenomics/GTDBTk

^11^ https://github.com/wwood/CoverM

^12^ https://github.com/DavidBSauer/OGT_prediction/

^13^ https://github.com/ParBLiSS/FastANI

^14^ https://web.expasy.org/translate/

^15^ https://github.com/lh3/seqtk

^16^ https://www.ncbi.nlm.nih.gov/books/NBK279690/

^17^ https://github.com/torkian/blast-QC

^18^ https://gtdb.ecogenomic.org/tree

^19^ https://github.com/rcedgar/muscle

^20^ http://www.iqtree.org/

^21^ https://itol.embl.de/

^22^ https://github.com/donovan-h-parks/CompareM

## Supplementary material

Supplementary Figure s1 Pathways for activation and oxidation of benzene in MAGs present with relative abundances between 3-5% in the benzene 50°C culture. For proteins and pathways encoded by several genes, completeness was calculated as percentage of present genes of total genes of the pathway/protein. For pathway genes and abbreviations see Supplementary Table 7.

Supplementary figure s2 Average nucleotide identity (ANI) between publicly availably DSM-4660 MAGs and DSM-4660 MAGs from this study.

Supplementary figure s3 Average amino acid identity (AAI) between publicly availably DSM-4660 MAGs and DSM-4660 MAGs from this study.

Supplementary figure s4 Average nucleotide identity (ANI) **(A)** and average amino acid identity (AAI) **(B)** between publicly available MAGs of the bacterial order SZUA-161 and of the SZUA-161 MAG, MAG 33, reconstructed in this study.

Supplementary table s1 Metadata on publicly available DSM-4660 MAGs included in this study. BioSample accession, and organism and isolate names were acquired from NCBI. Completeness and redundancy was acquired from GTDB-Tk. The optimal growth temperature (OGT) was inferred with OGT_prediction.

Supplementary table s2 Genome clusters of 135 DSM-4660 MAGs after dereplication at species-level (average nucleotide identity ≥95%), including representative genome selected by anvi‘o.

Supplementary table s3 Metadata on publicly available SZUA-161 MAGs included in this study. BioSample accession, and organism and isolate names were acquired from NCBI. Completeness and redundancy was acquired from GTDB-Tk. The optimal growth temperature (OGT) was inferred with OGT_prediction (see “Method” section).

Supplementary table s4 Calculations of doubling times of anaerobic benzene- and naphthalene-oxidizing cultures incubated at 50°C and 70°C based on sulfide production.

Supplementary table s5 Relative abundances of MAGs in UAH-degrading cultures obtained with CoverM and taxonomic classification with GTDB-Tk.

Supplementary table s6 Read counts per nearest taxonomic unit (NTU) of reconstructed 16S rRNA gene sequences in original sediment obtained with phyloFlash and relative abundances of MAGs from UAH-degrading cultures in original sediment obtained with CoverM.

Supplementary table s7 Pathways and genes involved in the anaerobic degradation of benzene and naphthalene.

Supplementary table s8 MAGs occurring with relative abundances of 3-5% in the benzene-oxidizing culture at 50°C. Optimal growth temperature (OGT) was predicted with OGT_prediction, taxonomic affiliation was determined with GTDB-Tk.

Supplementary table s9 ANI and AAI values of DSM-4660 MAGs.

Supplementary table s10 ANI and AAI values of SZUA-161 MAGs.
