# Supplementary figures and images for: Potential for the anaerobic oxidation of benzene and naphthalene in thermophilic microorganisms from the Guaymas Basin
Source: Front Microbiol. 2023 Sep 29;14:1279865. doi: 10.3389/fmicb.2023.1279865 (PMC10570749; doi:10.3389/fmicb.2023.1279865)

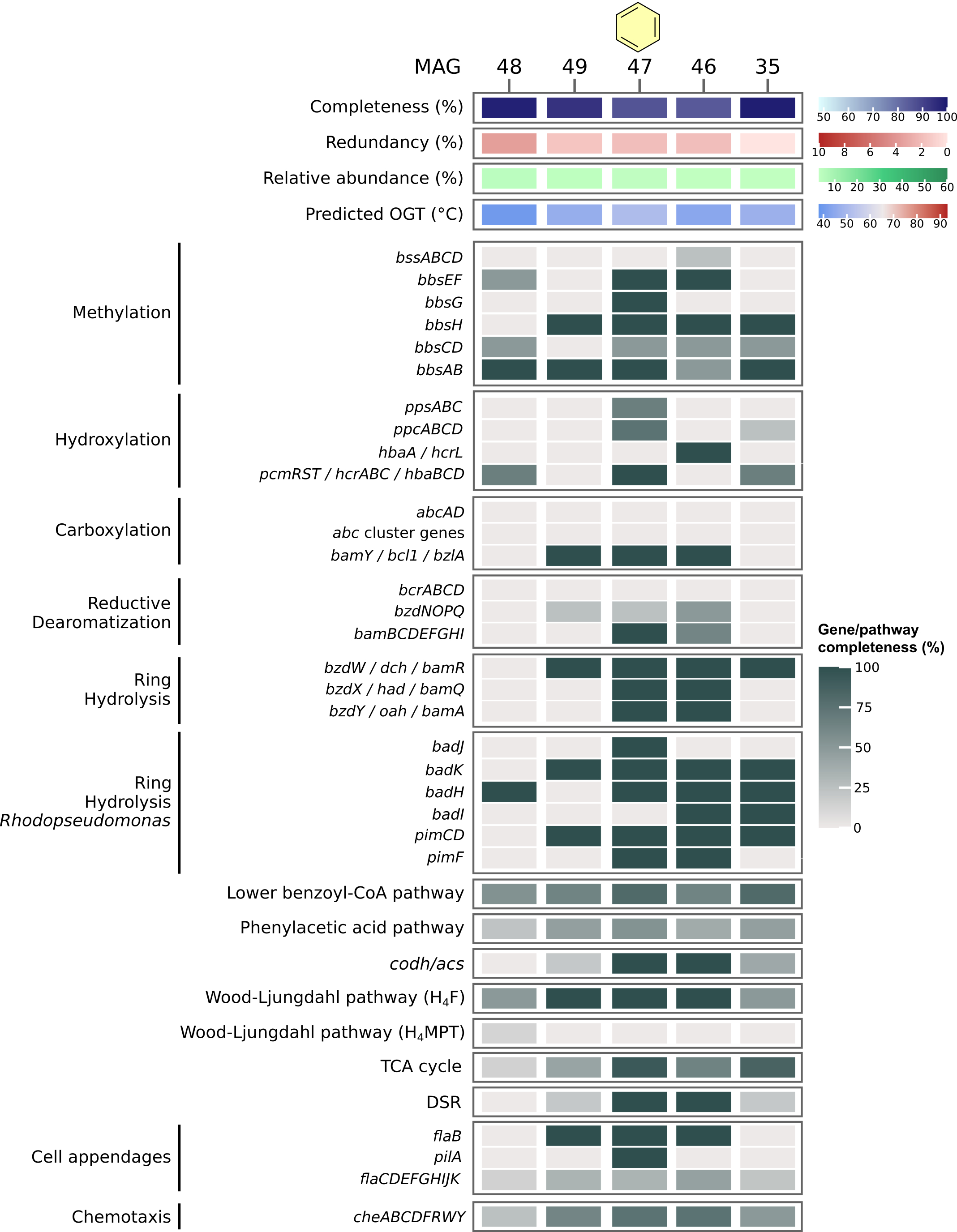

Supplement: Supplementary Figure s1 — Pathways for activation and oxidation of benzene in MAGs present with relative abundances between 3-5% in the benzene 50°C culture. For proteins and pathways encoded by several genes, completeness was calculated as percentage of present genes of total genes of the pathway/protein. For pathway genes and abbreviations see Supplementary Table 7. [file Image_1.JPEG]

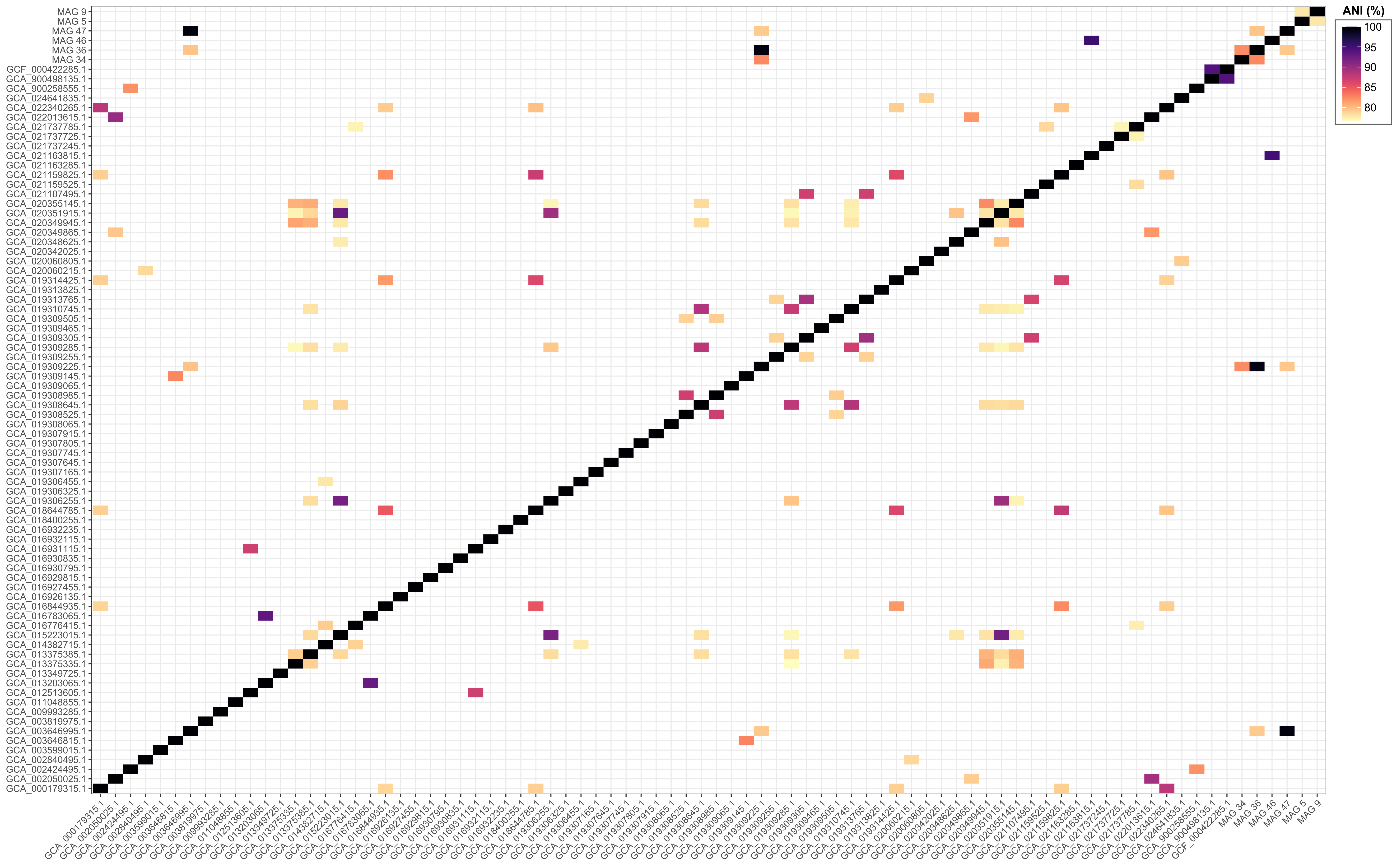

Supplement: Supplementary figure s2 — Average nucleotide identity (ANI) between publicly availably DSM-4660 MAGs and DSM-4660 MAGs from this study. [file Image_2.JPEG]

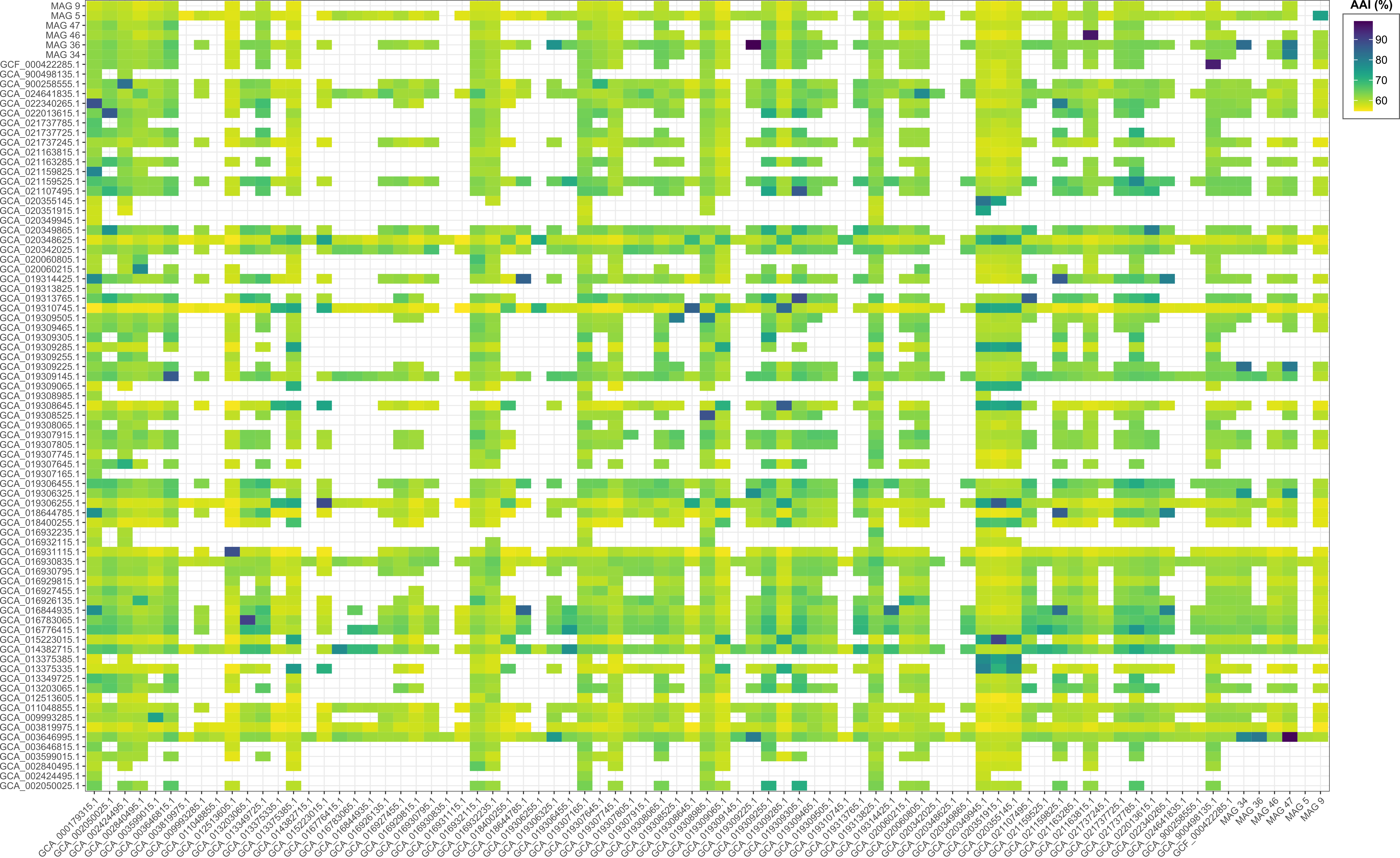

Supplement: Supplementary figure s3 — Average amino acid identity (AAI) between publicly availably DSM-4660 MAGs and DSM-4660 MAGs from this study. [file Image_3.JPEG]

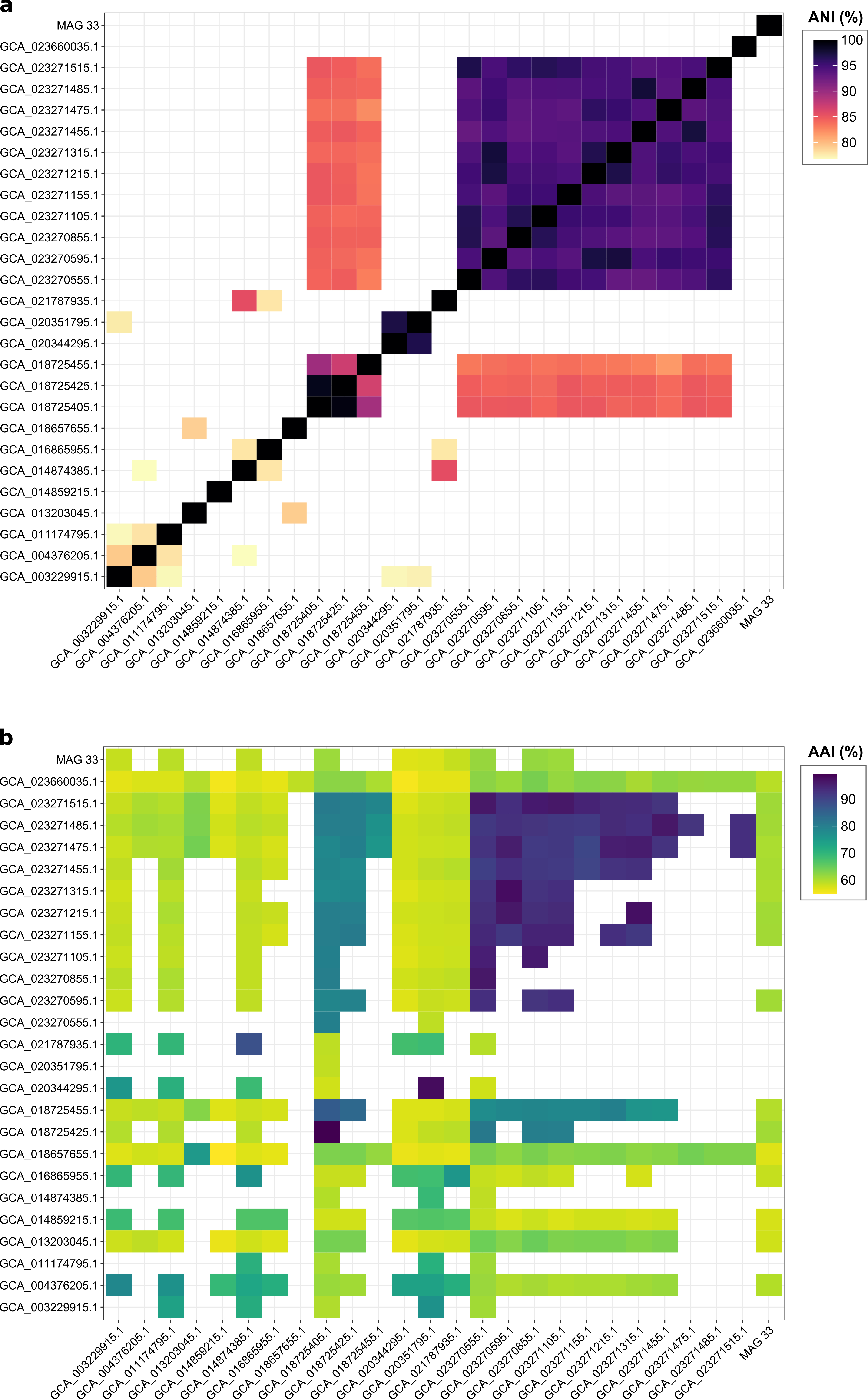

Supplement: Supplementary figure s4 — Average nucleotide identity (ANI) (A) and average amino acid identity (AAI) (B) between publicly available MAGs of the bacterial order SZUA-161 and of the SZUA-161 MAG, MAG 33, reconstructed in this study. [file Image_4.JPEG]
